# Understanding the Attitudes of Communities to the Social, Economic, and Cultural Importance of Working Donkeys in Rural, Peri-urban, and Urban Areas of Ethiopia

**DOI:** 10.3389/fvets.2020.00060

**Published:** 2020-02-14

**Authors:** Martha Geiger, Jo Hockenhull, Henry Buller, Gebre Tefera Engida, Mulugeta Getachew, Faith Adelaide Burden, Helen Rebecca Whay

**Affiliations:** ^1^Animal Welfare and Behaviour Group, Bristol Veterinary School, University of Bristol, Bristol, United Kingdom; ^2^Department of Geography, College of Life and Environmental Sciences, University of Exeter, Exeter, United Kingdom; ^3^Society for the Protection of Animals Abroad (SPANA), London, United Kingdom; ^4^The Donkey Sanctuary, Slade House Farm, Sidmouth, United Kingdom; ^5^Office of the Vice President International, The National University of Ireland Galway, Galway, Ireland

**Keywords:** donkey, livelihood, value, welfare, perception, attitude, working equids

## Abstract

Working donkeys (*Equus africanus asinus*) are vital to the development and support of people's livelihoods in rural, peri-urban, and urban areas of Ethiopia. However, despite their critical role in providing transport, food security, and income generation to some of the poorest and most marginalized households, donkey contributions to human livelihoods have been largely unexplored. Donkey users, veterinary surgeons, business owners, and civil servants were interviewed to investigate the role humans play in shaping donkey lives while furthering our understanding of the social and economic impacts of working donkeys to human lives. Findings are discussed through seven guiding themes; donkeys as generators of income, the relationship between donkeys and social status, donkeys and affect, empowerment through donkeys, the role of donkeys in reducing vulnerability and encouraging resilience, donkey husbandry, and gender dynamics all of which gave a broader and richer insight into the value of donkeys. Donkeys are an important support in rural, peri-urban, and urban settings through the creation of economic security, independence, and participation in local saving schemes. In addition, donkeys provide social status, empowerment to marginalized groups such as women and the very poor and provide a sense of companionship. Whether the interviewee was a donkey user or a key informant appeared to influence their views on donkeys and their welfare, as did their location. The variations in views and practices between urban and rural settings suggests that assessing the socioeconomic value of donkeys in different locations within the same area or country is critical, rather than assuming that similar views are held between compatriots. Despite their centrality to many people's lives in Ethiopia, working donkeys often hold lowly status, are misunderstood, and given little husbandry and healthcare.

## Introduction

Information on how working animals offer social, cultural, and economic (socioeconomic) value to their owners is critical for Non-Governmental Organizations (NGOs) and policy makers working with both human and animal stakeholders given that how animals are valued by societies can affect the animals' welfare status (1). However, information on socioeconomic value is inadequate or unavailable for many populations who employ working animals, meaning that decision making by these organizations often lacks a strong evidence base (1). This is particularly true for working donkeys which are commonly perceived to lack value in comparison to ruminant livestock (2). Consequently, the economic and societal contributions of donkeys to these populations are often overlooked, leading to them being ignored in initiatives developed by government policy makers (3–5). To address this situation and generate data on the socioeconomic value that working donkeys have for communities in different geographic locations, The Donkey Sanctuary worked in collaboration with researchers from the University of Bristol to develop and validate a tool that could be used with communities in a range of contexts to obtain standardized data on the socioeconomic value that working donkeys hold for these groups.

In order to develop a framework around which the tool could be based, the first step was to identify appropriate topic areas for the tool to encompass and thereby consider the concept of a donkeys' value beyond commonly applied, over simplistic constructs. To this end a qualitative approach founded on social science methodologies was adopted. Social science provides tools for examining sites where human-animal interactions occur and for understanding the human processes of valuing, caring for and treatment of animals (6, 7). To date, both natural science and social science methodologies used to understand the value and impact of working donkeys have generally consisted of gathering owners' own accounts of the donkeys' health status, self-measured livelihood reports, and researchers' assessments of the impact donkeys have on owners' income generation and work load (2, 8–11). While these methods are effective in generating information about the economic utility of donkeys (their contributions to peoples' households in terms of transport and household income), little is understood about the personal, social, and broader economic value of donkeys to rural, peri-urban and urban households.

The aim of this project was to identify what these broader values are through in-depth interviews with 30 key stakeholders comprising donkey owners, donkey users, and other key individuals working within a sample of communities in Ethiopia. Approximately 80 percent of the Ethiopian population lives in rural areas and earns a living from agriculture (8, 12). Working donkeys (*Equus africanus asinus*), are an important source of draft power and transport for many in both rural, peri-urban and urban areas throughout the year. In addition, donkeys are crucial to Ethiopia's growing economic landscape and to the social and cultural fabric of human society. Ethiopia has approximately 8.8 million donkeys, the largest population in the world (13). This creates a significant need to account for and represent these animals and their owners/users within the relevant literature and practical development discourse. Furthermore, Ethiopia provides an informative landscape for this initial exploratory stage of the wider project to identify broader values for inclusion in the subsequent tool for assessing socioeconomic value in working donkeys.

## Materials and Methods

### Ethical Approval

The study was approved by the Faculty of Medical and Veterinary Science Research Ethics Committee, University of Bristol (May 2014, Ref: 7502). Participant information sheets and consent forms were provided in the participant's first language of Amharic or Oromo and were also explained verbally in their first language to ensure comprehension prior to signing the consent form and starting the interview. Participants were given a culturally appropriate gift (coffee) as compensation for the time they had lost while being interviewed.

### Location

Data collection was carried out in 2014 in four locations in the Oromia region of central Ethiopia. The four interview locations were determined by the lead researcher and The Donkey Sanctuary Ethiopia and included the criterion that participants should not have been exposed to any previous or current equine charity work. This was to limit as far as possible the effect of outside influences that would have altered respondents' local knowledge, care regimens, and decision-making regarding their donkeys. The four locations selected were, the rural villages of Sululta and Ho-itu, the urban area of North Addis Ababa, and peri-urban Bulbula. The selection of the four interview locations was based on achieving a cross-section of donkey roles in order to interview and observe owners/users who were engaged in a variety of tasks and activities with their donkeys (described in detail in Table 1) to gain the widest range of perspectives as possible.

**Table 1:** Demographic information for the 20 donkey owners and users who participated in the study.

**Participant**	**Age range**	**Number of donkeys owned**	**Occupation**	**Location**	**Income generating task**	**Weekly income from donkeys (Birr)**
1	61–70	2	Farmer	Rural	Sale of dung for fire creation using pack donkeys	120
2	61–70	1	Farmer	Rural	Hiring out of donkeys; sale of dung for fire creation	70
3	31–40	2	Housewife	Rural	Transport and sale of firewood	140
4	31–40	1	Farmer	Rural	Sale of dung for fire creation	60
5	51–60	1	Housewife	Rural	None.	0
6	21–30	5	Construction Worker	Urban	Transporting water for sale; transporting construction materials	1,500
7	21–30	0	Construction Worker	Urban	Transporting construction materials	200
8	31–40	1 co-owned	Rubbish Collector	Urban	Collecting rubbish for government services	200
9	51–60	5	Shop Owner	Urban	Transporting construction materials	–^a^
10	61–70	13	Farmer	Rural	None.	0
11	21–30	7	Farmer	Rural	Transporting people to various locations	100
12	41–50	5	Farmer	Rural	Transporting people to various locations	120
13	61–70	1	Farmer	Rural	Transporting and sale of grain at markets	0
14	31–40	4	Farmer	Rural	None.	0
15	31–40	2	Farmer	Peri-urban	Transporting and sale of grain at markets	0
16	41–50	1	Shop Owner	Peri-urban	Transporting materials for sale from urban to rural	–
17	41–50	1	Farmer	Peri-urban	Transporting materials for sale from urban to rural	–
18	31–40	1	Water Distributor	Peri-urban	Transporting water for sale	200
19	51–60	1	Cart Owner	Urban	Harvesting, transporting and sale of crops; transporting materials for sale from urban to rural; transporting furniture	600
20	41–50	2	Farmer	Peri-urban	Sale of dung for fire creation	50

a *The income information was not provided by the participant because the amount was claimed to be unknown*.

### Sample

Twenty primary donkey owner/user and 10 key informant in-depth interviews were conducted in total. Donkey users are classified as persons who do not own their own donkey but used donkeys through renting or borrowing from other community members. The sample consisted of 17 men and 13 women, with an even split of 10 female and 10 male donkey owning/using participants and seven male and three female key informants. Key informants consisted of community stakeholders such as veterinary surgeons, business owners, police officers, and village chiefs. There were limitations on gaining equal gender representation within the key informants as it proved to be challenging to find enough women holding professional positions within the communities where the research was conducted. Five primary interviews and two or three key informant interviews were conducted in each location.

### Recruitment

Recruitment of participants involved engagement with the Rural Development Agents who worked in each rural location to assist the research team with introductions to potential participants and the wider community. The Rural Development Agents were provided with the sampling criteria (e.g., veterinarians, donkey owners, donkey users, business owners etc.) and translated letter of information detailing the aim of the research study in order to assist them with the introductions to potential participants and the research team. However, in the urban and peri-urban areas, the research team directly approached people they encountered who were working with their donkeys to recruit them for an interview as there were no Rural Development Agents working in those areas.

An experienced local assistant interviewer was hired to conduct the interviews in the local languages, Oromo and/or Amharic. The assistant interviewer had previous experience as an English translator, had conducted interviews on working equine-related research studies, and held a degree in sociology and a diploma in veterinary medicine. To support the assistant interviewer's qualifications, training was provided by the lead researcher regarding the objectives of the study and the structure of the interviews.

### Interviews

**Figure 1:**
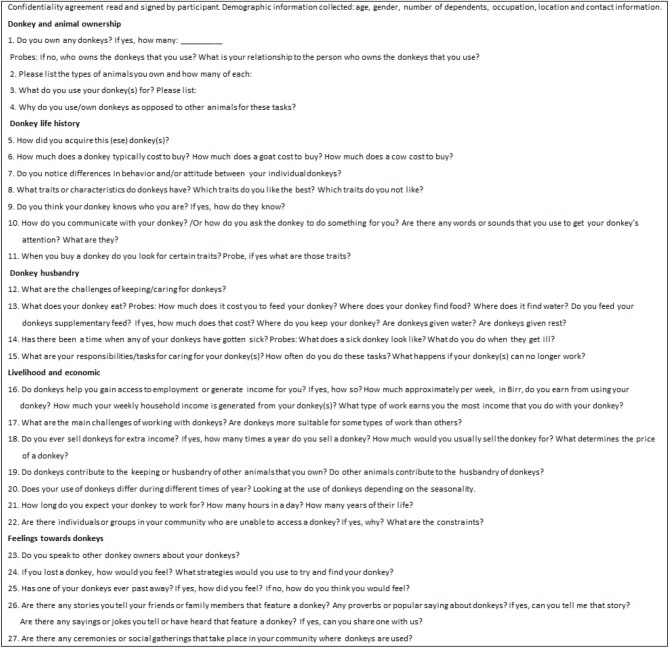
Interview Guide for donkey owners and users.

Prior to data collection, an interview guide was developed with a set of questions and areas the research aimed to explore to assist the researcher and assistant interviewer in shaping and focusing the discussions with the participants during the interviews. The interview themes and questions were developed based on previously identified gaps in the literature on the socio-economic impacts of working donkeys in the global south. The key themes and questioning guiding the interviews consisted of donkey husbandry, economic contributions, social contributions, ownership history, utilization, and perceptions of donkeys in Ethiopian society. The methodology allowed the researcher to go beyond the answers that the interviewee believed the interviewer wished to hear. The semi-structured interview guide is provided in Figure 1.

One pilot interview was conducted in the first location prior to data collection to ensure the questions were clear and that the themes were relevant to the context. The transcript of the pilot interview was assessed, and necessary changes and additions were made to the interview guide.

The interviews lasted between 1 and 2 h. After the completion of each interview, the assistant interviewer and researcher would discuss and document their own observations to check consistency in understanding. When each interview location was completed, the assistant interviewer would play back the encrypted mp3 audio recorded interviews and translate verbally into English, while the lead researcher would transcribe and ensure clarification on relevant subject matters. Each transcription was encrypted and anonymised.

### Analysis

After the completion of the data collection, a qualitative content analysis of the interview transcripts was undertaken. The analysis began by reading through the transcriptions for re-familiarization and to comprehend essential features of the content. The data were then organized into categories to build a coding framework that was subsequently divided into major themes and subthemes using descriptive and thematic coding (14, 15). Once the quotes from the participant interview transcripts were organized into respective categories by systematically identifying specified characteristics of each category, a quantitative analysis was performed in a separate database where interviews were carefully scanned for how often participants shared similar sentiments, ideas, experiences, or opinions. The frequency was then documented for each characteristic and the key themes and concepts were then summarized.

## Results and Discussion

Seven key themes and concepts emerged from the analysis of the interview transcripts. These are discussed individually below.

### Donkeys as Generators of Income

The donkey owners, users, and key informants who participated in the study varied considerably in terms of occupations, ages, donkey access, and income levels, providing a breadth of information on the multifaceted ways donkeys' impact peoples' livelihoods (Table 1). Income-generating activities performed by donkey owners differed across rural and urban settings; however, the completion of the majority of tasks involved donkeys and their owners traveling between urban and rural settings (Table 1). The two most common uses of donkeys in the four locations were pack-carrying and cart-hauling. However, observations were made of younger children and youth riding on donkeys in the rural areas. The most common income generating tasks carried out by owners/users and their donkeys were the sale of dried dung at markets as a source of fuel for cooking fires, the transport of materials for sale in urban and rural areas and using cart donkeys to harvest, transport and sell crops. The tasks reported by study participants are comparable to those reported in previous studies (16).

It is clear that the donkeys' work enabled their owners/users to generate income. However, donkey ownership and the work they do was also used to achieve a degree of security for people who live in vulnerable and impoverished situations. Individual donkey owners/users were each found to have individual spending, insurance and investment strategies for using the income earned through their donkeys' work. The income generated by participant donkey owners/users was typically spent or saved for family or household needs such as buying food stuffs from the market, purchasing additional livestock, or equines, buying school materials for children, repairing or building homes, and/or participating in the weekly or monthly community based saving schemes called *Iddir* and *Iqqub*. Iddir is a traditional community savings, credit, and insurance cooperative that is designed with the main goal of providing members and their families with assistance in the event of death, illness or unemployment or for member weddings (17). Iqqub is an informal savings and credit scheme whereby members contribute a fixed amount of money weekly, bi-weekly or monthly, the Iqqub sum is then given in rotation to the participants each collection time (17, 18). Iqqub and Iddir can help participants purchase animals, including donkeys, and build and repair homes. Participants in Iqqub or Iddir schemes who have lost a donkey will receive financial compensation to purchase another donkey. In addition, Iqqub or Iddir provides reassurance and financial support to the wider community in emergencies or in times of particular hardship. This type of traditional, local financial system acts as an important saving, and insurance scheme for community members. These systems are accessible to any community member who can regularly contribute the required sum of money and are available to those who are unable to access formal banks or insurance programs. The sums vary between regions and communities, however, according to Tadesse and Brans (17) urban workers can individually contribute 200 ETB per year minimum and agro-pastoralists can contribute a minimum of 50 ETB each per year. For further context, the average monthly wage across Ethiopia is 1,305 ETB or $44 USD (12). Gaps between urban and rural poverty are large, with 25.6% of the rural population living on or below the poverty line and 14.8 % in the urban areas demonstrating the difference between the financial capabilities of people able to contribute to programs such as Idir or Iqqub (12). Donkey owner participant number 18 explained how she uses the Iqqub money she receives when it is her turn:

“Generally, I will purchase a large or small animal when I get Iqqub money 2–3 times per year and then I will get them [the animal purchased] fatter and sell them for a good profit” (June 19, 2014).

For context, owners/users from the field sites referred to donkey body condition status as “fat or skinny.” Skinny donkeys were viewed as less desirable for work because they are seen as “less healthy” in comparison to donkeys who were “fatter” and more desirable because their body weight was viewed as an indication of better health and ability to work. These traditional community-based systems, where each individual relies on the other members for assistance and participation, help community members build assets, and insure themselves against unforeseen shocks to their community. Donkeys are an important pillar for these informal systems because, as cited by participants in each location, they are the main enabler for the owner to generate an income and participate in the scheme. Donkey owner number 4 explained:

“I am feeling happy every day when I am getting benefits from my donkey, but my husband died a year ago, so it is only me who is earning an income. I feel happy whenever I pay every week or every 2 weeks for Iqqub or Iddir because I realize that if I couldn't get service from my donkey I can't pay into either and won't get help from these organizations” (June 9, 2014).

As this quote illustrates, donkey owners/users can create stability in their lives through participation in these community-based saving systems by using donkeys to generate income. The donkey performs a series of quite different economic functions for those that own and use them. The importance and value to the individual owner/user is displayed by the intrinsic feelings of happiness and relief this participant expresses at having her donkey and being the sole provider for her family.

Not only do owners benefit from owning and working with donkeys, the wider community benefits economically in several ways: when community members are unable to own donkeys either through lack of land access, lack of money, or lack of personal capacity, donkey owners will often let these members borrow their donkeys for certain days or tasks free of charge or rarely for a small fee. In Tadesse and Brans (17) study in pastoral areas of Ethiopia, households that have suffered a loss negatively affecting access to milk and butter will borrow a cow from a community member until the household has had time to recover. In the event of a larger shock such as a large loss of livestock, the community will contribute animals from their own herds to help that household recover (17). Similarly, donkey owners will lend non-donkey owners their carts to assist them in generating income or completing their tasks. However, carts are costly to buy and are perceived to be more costly to maintain and repair than donkeys, so owner/user participants explained that they were more likely to charge someone a fee for using their cart. Often they would not let people beyond their family circle use their cart because of the financial implications, such as the expense of replacing a tire. Research on the use of micro-insurance in Ethiopia reveals that those who are the poorest of the poor are excluded from these schemes due to lack of funds required in order to participate (17). This finding supports the participant's claims that donkeys are a pathway out of extreme poverty and provides them with enough income generation that allows their participation in these savings and credit schemes.

### The Relationship Between Donkeys and Social Status

Donkeys are important economic contributors and assets, but they are also important to the social fabric of human life in rural and urban communities. Donkeys provide owners with empowerment through independence, status, employment, health, and happiness; however, the donkeys' assigned roles in Ethiopia can perpetuate societal inequality such as being viewed as ‘a poor persons’ vehicle'. Donkeys' very nature (their ability to survive drought, their physical strength, and their perceived stoical nature) can also potentially affect their own wellbeing; these characteristics result in societal perceptions that can encourage neglect (7).

While eighty percent of donkey owners/user participants reported having greater security against environmental and financial hardships, community members who were not donkey owners, horse owners or vehicle owners were seen by others as having the lowest economic and social status who were at risk of serious deprivation, as expressed by donkey owner participant number 12:

“I speak to my community members about the benefits of having donkeys and I advise people to buy donkeys. People who do not own donkeys are under-privileged, not respected and underestimated; they are living in poor conditions. They are the poorest sector of the community. Those who don't have donkeys are in definite poverty. I advise such people to buy donkeys and use donkeys to get themselves out of poverty. People will help them for a while but finally they will reject them forever” (June 17, 2014).

For many smallholder farmers in Ethiopia, financial capital is often measured through ownership of livestock (19). At the same time, social capital is formed and strengthened through relationships with other community members and their shared interests in livestock (19). Social capital refers to the “norms and networks that allow people to act collectively” (20). Social capital built upon mutual community trust and strong social ties is exemplified by the Iddir and Iqqub schemes described earlier. Since social capital and community economic security are built and strengthened through the ability of the community members to assist one another, the non-donkey owners risk being shunned. Non-donkey owners who continue to borrow donkeys for longer lengths of time risk being viewed as dependents, in contrast to those who borrow donkeys for an interim period to earn enough money to buy another donkey; this latter group are not viewed as dependents but as ones who have fallen on hardship. This type of borrowing arrangement of livestock after a shock or loss has been supported by micro-insurance research conducted by Tadesse and Brans (17) and Tadesse (21).

People who are able to afford to buy strong, healthy young adult donkeys may be the ones who are able to carry out the most livelihood and economic enhancing tasks (e.g., ploughing, transporting goods for sale, or owning a construction business). In contrast, those who are unable to afford a donkey with the most preferred traits may not be as prosperous. People who are unable to afford the most preferable donkeys are only able to purchase very young donkeys, not fully grown, or donkeys with unfavorable physical issues such as wounds, limb deformities, poor body condition, or lameness. Those who have limited available money to purchase a donkey may have to resort to buying a donkey that has visible physical issues, behavioral issues or that is older in age and may not have the working life expectancy that younger, healthier donkeys may have. This disparity marginalizes those in communities who live in greater poverty relative to other members. Therefore, the welfare of both the donkey and the human could be compromised. Geiger and Hovorka (22) found in their 2012 study similar connections between donkey and human wellbeing whereby donkeys are unable to fully assist people in securing their livelihoods if the donkey's welfare was compromised. In conditions where hardships are common and income levels are low, it is important for the community to work together to support one another, to achieve financial and social stability. People such as single heads of household, widows, unemployed persons, the sick and the elderly may have less access to donkeys that are in appropriate working condition, for example donkey owner number 3 explained:

“Female headed and single women households find it difficult for them to purchase donkeys and use them. Sometimes we support them and give them donkeys, the community members” (June 9, 2014).

Participant sentiments support Tadesse and Brans (17) findings of community livestock gifting and lending during times of hardship and indicates that donkey owners share their knowledge through word of mouth, emphasizing to non-owners the importance of saving money to buy donkeys and other livestock. Such advice is also given to younger members of the community; children are encouraged to save money and look after the donkeys and other animals they own. Donkey owner number 17 tells his children that:

“Someone who does not have donkeys are the poorest ones; donkeys are giving help all throughout the year. I tell my children to care for animals with four legs because it may take 4 years to acquire a four-legged animal. It is the cost of saving the money. If you want a chicken you could buy a chicken if you work hard for 2 days. But if you want to buy an oxen or donkey you have to work for 4 years; this is what the elders said to me. I advise my children this way and when I want my children to care for my animals” (June 19, 2014).

However, the main constraints cited by fifty-five percent of participants regarding access or ownership of donkeys was not the length of time to save up to buy a donkey or the lack of assistance in caring for donkeys; the two main constraints preventing people from owning donkeys across the four study sites were perpetual poverty and lack of access to land, as explained by donkey owner number 5:

“There are some people who fail to use donkeys because they don't have money to purchase a donkey and I help these people by letting them use my donkey free of charge. The donkey is too expensive for people to buy. Some people do not have land to keep donkeys and no place to let them graze. There is land scarcity” (June 9, 2014). This is particularly the case in urban settings.

Previous studies in Ethiopia have also highlighted lack of grazing land as a limiting factor for donkey ownership (16, 23, 24). Despite these constraints, participants in the urban and peri-urban sites maintain that non-ownership of donkeys may also be linked to a lack of knowledge about how to use, care for, and work with donkeys. Participants explain that in general those who moved from the rural areas or live part-time in the urban areas are the ones who most often own donkeys. Those who live partly in the urban areas to earn an income are able to return back to the rural areas with the transport from their donkeys.

### Donkeys and Affect

Seventy percent of donkey owners/users thought and spoke about their lives and the work they do with their donkeys expressed feelings of happiness, comfort, security and relief at the alleviation of the demand for excessive labor on their part. Owners/users appreciated the animals' assistance with daily tasks and the strength that enables donkeys to work long hours throughout different seasons. Donkey owner number 18 described the feelings of joy and security she gets from owning and working with her donkey:

“I am always happy doing this business with my donkey. This donkey is the base for helping my family's life. All my family's income is from this donkey, so I am always happy using my donkey” (June 20, 2014).

Feelings of happiness were commonly expressed when participants explained the way they value their donkey and how they feel when working with their donkeys. Donkeys were described by participants as “friends for life.” Gratitude for the support donkeys provide was also a common sentiment when owners/users spoke about their donkeys. These sentiments indicate the deeper meaningful importance of donkeys to their owners/users beyond their financial or work contributions; these sentiments afford a glimpse into how people conceptualize their relationship with their donkeys. For example, donkey owner participant number 6 expressed feelings of grief and loss when his donkey passed away:

“I can't tell you what happened, I saw that my donkey couldn't eat and died. I felt as if I missed one of my friends because donkeys are my source of income, my source of life” (June 13, 2014).

Sixty percent of donkey owners/users recounted personal stories of grief and sorrow over the death or loss of a donkey; although this loss or fear of this loss was often accompanied by concern over not being able to access the market for selling and purchasing household items, not having transport or being able to complete tasks. Although superficially opposing, affective, and instrumental perceptions of working equines are recognized to coexist (25). Donkey illness or death can have a negative emotional impact on the individual who owns and/or works with the donkey and can adversely affect the family financially. It can often be difficult to distinguish between the two, to untangle the emotional and practical response to the loss. In addition, if communities lose donkeys, they also lose people who are able to participate and contribute to community-based programs such as Iddir or Iqqub. For example, donkey owner number 4 explained the donkeys' importance to income earning and daily life activities and indicated the emotions that are felt when a donkey is ill:

“Sometimes the donkey gets sick while working with them. Sometimes they can't urinate… Just a few months ago he couldn't urinate. I feel very sad when my donkey gets sick because I can't get the grains to the market and won't earn any income when my donkey is sick” (June 9 2014).

However, donkeys could also generate negative feelings besides sadness. These consisted of anger and frustration toward the constraints that prevent donkeys from performing their assigned tasks. For example, donkey “misbehavior” can cost owners/users in time, loss of resources such as market purchases or loss of money through replacing ruined goods such as spilled cement or grain bags. Donkey owner number 7 expressed his upset as follows:

“When donkeys run away when they are being loaded I get angry with them, that sometimes happens, generally they are good in behavior. Donkeys will also run away when they are getting lazy and weak, but that is part of the business at the end of the day they refuse to work because of the burden of that day. Usually, they force them to work” (June 13, 2014).

This quote not only displays negative feelings toward donkeys themselves but also demonstrates the tensions that can occur between donkeys and their owners because of the heavy physical exertion and dependence on the donkey to perform the work in order to ensure their livelihoods.

### Empowerment Through Donkeys

Participants also described the importance of having donkeys in gaining independence and being able to begin to create opportunities for employment and/or begin their own business using donkeys for transporting materials to markets or in construction areas. This gave owners feelings of empowerment through working for themselves with the assistance of their donkeys. Donkey owner number 6 conveyed this when describing the change in his life when he stopped working for someone else and got his own donkeys:

“I used to work by being hired by someone else before and I felt very glad and happy when I got my own donkey for the first time and started earning from my own donkeys. I felt free by earning my own money and being free of anyone else and earning my own income with my own donkey. I felt so happy and independent. I will always remember that time in my life” (June 11, 2014).

Through empowering individuals by enabling them to earn a greater income and to reduce their reliance on others, donkeys have a pivotal role in improving social status, self-esteem and independence and therefore potentially enhancing people's quality of life.

All thirty participants reported that donkeys performed vital livelihood tasks. Participants explained donkeys assist in delivering water and the sale of water to water-poor areas, transport firewood for sale, provide access to areas where motorized vehicles cannot reach, generate business opportunities, and help people access far-away markets during times of famine and drought; all of these were cited as very important tasks the participants were able to complete because they owned a donkey. Donkey owner participant number 10 explained the importance of donkeys during periods of drought:

“In the 1980s during the drought, we [his family] were trading with donkeys because there were no grains available. We bought grains from very far distances; we would transport them with our donkeys and sell in our village and change the grain for money. At that time donkeys were very useful; if donkeys weren't used at that time too many people would have died. Donkeys are drought-resistant” (June 17, 2014).

Donkeys are seen to be very hardy animals that are easier to keep than horses and oxen, similar sentiments have been documented by donkey owners/users in Geiger and Hovorka's (7) study looking at donkey welfare and lives in Botswana. One donkey-owning participant recalls during a longer period of drought, donkeys were given to communities to help with transport and trade when many people were losing their livestock because of feed and water shortages. Participant number 10 explained:

“There was a time when drought happened and donkeys were given to the communities by the government to help them with trading and transport. Equines are recognized by the government as transport animals” (June 17, 2014).

While donkeys are claimed to be recognized as important animals to the agricultural sector, their current inclusion in livestock and food security policy frameworks is poor (26, 27). In fact, donkeys are not included in any livestock development programs or policies in Ethiopia. Key informant number 8 and pharmacy owner explained that donkeys are the equivalent to camels for communities because they can survive droughts when sheep and cattle cannot. He explained:

“Other animals cannot resist drought. So for this community donkeys act as camels…Donkeys are very strong, stronger than other animals so the community should be aware of this” (June 19, 2014).

The value of donkeys apparent in sentiments like these reflect the way that donkeys can create security for their owners and are animals that can be relied upon by their owners and communities during difficult events.

### Donkey Husbandry

The perception of donkeys as particularly hardy animals could have a negative impact on the donkey insofar as the perception of hardiness can result in them receiving a diminished amount of care and attention and an unrealistic view of the amount of work they can do for their owner. Donkey owner number 9 explained a common perception held by some owners, users and community members viewing donkeys as strong, hardy animals that can endure harsh treatment and hard work:

“People usually say that donkeys are very strong, and donkeys are an animal that resist many hardships. That's why when people hit each other, and people beat each other with sticks people would say ‘wow he beat him like a donkey’. It means that donkeys are okay and will still be ok when being beaten or being hit dangerously” (June 13, 2014).

Thirty percent of donkey owners/users expressed their view that some donkeys can be lazy. Donkeys who may be perceived as lazy could actually be misinterpreted by owners/users when they are actually tired, in pain or poor health and no longer able to work at their former pace. This societal perception could result in aspects of their health and welfare being neglected. For example, donkey owner number 17 described the difference between a lazy donkey and an alert donkey:

“Donkeys who are lazy, who are not alert cannot work for longer times. Alert donkeys will run quickly without being beaten but lazy donkeys need to be beaten with a stick to walk and that's why most people prefer alert donkeys” (June 19, 2014).

Thirty percent of participants held the perception that donkeys require physical force such as hitting or beating when handling them to motivate them to work when they are perceived to refuse. This also coincides with the owner/user management practices of the donkeys' when they exhibit perceived “bad” behavior. One owner explained they purposely restrict or limit food given to donkeys because when their donkeys' eat a lot and become healthy and more energetic they reportedly refuse to work and/or run away. If donkeys are too difficult to manage, people struggle to work with them and will not be able to complete their tasks. Owners/users indicated that if a donkey is too strong and aggressive, they will not be able to work with them. Donkey owner number 2 explained what he does with his donkeys if he experiences difficulty:

“Donkeys are good animals to load and use but if they refuse to be loaded we will sell them. Sometimes they get stronger and stronger and refuse to be loaded and then we will sell them” (June 6, 2014).

Donkeys, as explained by donkey owner number 2, are thought to be naturally strong and require little care, unlike other working animals such as horses and oxen that are perceived to require more feed supplements and maintenance like vaccinations or grooming. Management of donkey husbandry appears to be more focused on achieving the needed tasks donkeys perform rather than meeting the donkeys' physiological needs. Eighty-five percent of owners/users reported feeding their donkeys available grain or purchasing grain on the days they are needed for work, but reported being unable to consistently afford to feed their donkeys throughout the year. Throughout much of the year, donkeys subsist by grazing in nearby rural pastures, fields and grassy areas (in urban settings).

### Gender Dynamics

Forty-five percent of donkey owners/users cited donkeys as being most important to, and most valued by women, especially women living in rural communities. Donkeys greatly assist women in the tasks they typically perform within the gendered divisions of labor, such as transporting water from wells to home, collecting firewood, transporting cow dung to markets to sell, and transporting grain from market to home or home to market. In addition, responsibility for the provision of care for the donkeys is typically assigned to women within families. Women who do not have access to donkeys explain that their daily tasks are made more physically demanding as they have to carry heavy loads of firewood and other materials on their backs. Key informant number 1, a community health care worker, illustrated the importance of donkeys to women's lives and their responsibility for providing care to donkeys by stating:

“If there are no donkeys at home it is women who are carrying everything on their backs. So it is women who focus on donkeys. If you compare, it is women who mostly use the donkeys and focus on the donkeys. Women overall use donkeys more than men” (June 6, 2014).

This supports the participant observations made during the data collection period, when women carrying large loads of firewood, grasses, and water on their back to markets and homesteads in rural and urban areas were frequently observed. Curran and Smith (28) study which explored the economic contribution of donkeys to households living in peri-urban areas of Addis Ababa, found that donkey owners expressed feelings of relief from the burden of carrying firewood and water. Participants also reported longer term gains from donkey ownership including children being released from their daily chores, and therefore having the opportunity to go to school (28). Participant number 20 explained the differences in her life from not owning to owning a donkey:

“Before I owned donkeys I was carrying water and grain on my back and sometimes I would borrow a donkey from my neighbors. I felt very tired carrying water and grain on my back so I prefer to use donkeys. It is better because I was feeling tiresome carrying everything on my back, but now that I have my donkey I am feeling good. It was very tiring work in the past because some parts of the path on the way to the market are hilly and when I was carrying cow dung on my back I was feeling very tired” (June 20, 2014).

Women are also impacted emotionally by the loss or injury of a donkey. Women are seen to have closer relationships with donkeys than men, using them for a wider variety of tasks and because it is women who care for them and handle them on a daily basis. Key informant number 8, a pharmacy owner, explained:

“People feel sad when donkeys die, especially women, women cry when donkeys die. Donkeys are special animals for women to create businesses with, use for transportation purposes and they will not feel as sad when other animals die. Most animals in the family are more friendly toward women because they feed them, they care for them, they give them water, and they use them. Donkeys are not friendly with men. They are not intimate with men. Women are friendly with animals because they spend most of their time with animals, they worm them, handle them, spend their time loading, unloading and milking them. That's why their relationship with them is intimate” (June 20, 2014).

This quote reveals the physical, economical and emotional value of donkeys to women; their donkeys are close and important friends. It is apparent that women and men are both impacted by donkeys through the empowerment they experience through the use and ownership of donkeys that can create positive economic impacts to their lives through independence, protection from certain financial and physical vulnerabilities, and increase in social status within communities. Both men and women participants spoke of being emotionally impacted by donkeys through experiencing a loss of a donkey or the excitement of owning their first donkey and therefore may equally have close affective relationships and bonds to their working donkey(s), thus displaying the importance of the role of working donkeys to both men and women's lives. Yet, despite this, donkeys and women are generically marginalized by their status in society. A common proverb told by four participants was, “women and donkeys are the same-they both like to be beaten,” illustrates the alignment of the status of donkeys and women as being undervalued, under recognized and mistreated. The responsibility of care for the donkey(s) are often assigned to women, reinforcing the idea that women and donkeys are closely linked in terms of treatment and marginalized social status in comparison to those who earn higher incomes outside the home, such as men and cattle. However, societal perceptions, attitudes and treatment toward both women and donkeys is a connection that has yet to be fully explored. Improving the status and treatment of women may well-lead to improvement in the treatment of donkeys. Therefore, further investigation into this relationship is needed to understand how human and donkey welfare is connected and the implications of the relationship for animal welfare.

Participants also reported biases and stereotypes associated with donkey gender and used these to describe their preferences for one over the other. When asked if there was a gender preference when it came to donkeys seventy percent reported a preference toward male donkeys. Donkey owner 3 explained:

“Yes, there is mostly male donkeys are preferable. Why? Because female donkeys are always pregnant and during breeding season many male donkeys come to the compound and destroy all the compounds. That's why people prefer male donkeys” (June 9, 2014).

Health extension worker, key informant 1, also reported a preference for male donkeys, although he cited a different explanation:

“It is the male donkeys who have a higher price. The male donkeys carry more things than the female donkeys” (June 9, 2014).

Practical reasons were also given by animal science development agent, key informant 2, to explain why she preferred female donkeys:

“I think female donkey behavior is good, but male donkeys run away often. Female donkeys are fine they don't run away. The male ones run often. I like the female ones because they are comfortable to handle for me” (June 6, 2014).

It was suggested that donkey gender preference differed between rural and urban settings, as key informant number 4, an urban security guard explained:

“In this area using female donkeys isn't common because people don't want to use female donkeys because they can't work. People use them for breeding in rural areas but not in Addis” (June 12, 2014).

### Differences Between Urban and Rural Settings

Throughout the interview transcripts, a number of differences in practices, values and perceptions were observed between participants occupying rural and urban areas.

Participants explain that it is difficult to keep donkeys in the urban areas because of lack of grazing land, appropriate shelter, and the knowledge and means for general donkey husbandry. Clearly, further research is warranted into the condition of donkeys in urban areas: they may be fewer in number but, individually, they have more people depending on their work than in rural areas; do they, as a result, also have more compromised welfare? Welfare issues as observed and cited by owners/users and key informants in urban areas included injury and loss of donkey life via traffic accidents, health problems, inappropriate equipment, over-working and over-loading, chronic pain and lameness, insufficient provision of food that leads donkeys to seek food at rubbish sites, where they consume inappropriate food stuffs, and lack of available veterinary care. Lack of care will eventually limit the productivity of the donkey and restrict the amount of work people can do with that donkey.

In both peri-urban and urban study locations, participants explained that landlords would rent out space to house donkeys to as many donkey owners as they could, often leading to overcrowding. According to veterinary surgeon key informant number 3, such practice enables the transmission of infectious diseases, as well as creating conflict among donkeys and possibly injury. Urban landowners were cited as profiting most from the presence of donkeys: they earn income from the sale of the dried dung left in their shelters at local markets for use as fuel for fires. They also benefit from the work of donkeys indirectly: they can generate monthly income by renting out shelters or enclosures for people to keep their working donkeys overnight. Two participants expressed their concerns with these rental arrangements, citing the lack of management and monitoring of these rental shelters. Donkey owner participant number 6 explained that he has a compensation arrangement with the owner of the rental shelter where he keeps his donkeys overnight. If a donkey is injured overnight in the shelter the shelter owner will have to pay compensation. This is a mechanism for preventing loss or injury to the donkey and an effort on the part of the donkey owner to ensure the donkeys have a safe and comfortable place to stay at night.

This contrasts with the overnight management of donkeys in rural areas as Key informant 2, a development agent in animal science explained:

“People keep their horses and donkeys closer to their home in a separate enclosure because donkeys are vulnerable for hyena, the rest of the animals stay outside” (June 6, 2014).

Welfare issues affecting donkeys in rural areas were not mentioned as explicitly as they were in urban locations. The main concerns cited for rural donkeys were the tendency to overload the donkeys, the abandonment of ill or dying donkeys to hyenas and the necessity of keeping donkeys safely enclosed at night to prevent hyena attack. Participants in urban locations had the perception that rural donkeys worked less than those in urban areas and this may be why fewer welfare concerns were raised for rural donkeys. The abandonment of ill or dying donkeys when actions taken to resolve the problem were not successful seemed accepted practice in some rural locations. Commonly mentioned ailments affecting the donkeys included the inability to urinate and colic. Ill donkeys were typically treated with traditional cures such as local herbs in saltwater, chillies inserted in the donkey's nose or throat or a silver ring inserted in the urethra. While professional help may be sought for treating ill livestock, the same service was sometimes not sought for donkeys. Donkey owner number 3 explained that this was not something people did in her area:

“People may laugh at us if we take the donkey to the clinic people will think we are taking the donkey to kick the doctor. They will laugh at us” (June 9, 2014).

However, in other rural areas participants did report seeking professional help for their donkeys and continued to look after them once they could no longer work rather than abandoning them:

“We never ever throw a live animal out overnight, if we do that god will punish us” (Donkey owner number 10, June 17, 2014).

There was a difference between the levels of dependency on individual donkeys in the urban vs. rural areas and individual dependency on donkeys may also transfer when they move to different locations, as Donkey owner number 16 explained:

“In the rural areas everyone owns donkeys and there are enough donkeys for the community, but the urban area is not comparable. Many people are living in urban areas and not using donkeys, but some people are using donkeys for income. There are few donkeys helping many people in urban areas because people do not always use donkeys and are not involved in farming. Many people do not have any knowledge of donkeys and that's why they don't want to have donkeys. People who come from rural areas to urban areas are the ones who have donkeys” (June 19, 2014).

### Differences Between the Views of Key Informants and Donkey Owners/Users

It was interesting to note that the views of the Key informants differed greatly at times from those of the donkey owners/users. Often the key informants expressed derogatory opinions of donkey owners/users' practices in relation to their donkey. For example, key informant 1, a health extension worker, explained:

“I think people are not taking care of their donkeys properly. [She laughs about the question of why people aren't taking care of them]. In this area people keep their horse outside of their home, they consider the donkey doesn't need a lot of food …. They don't feed donkeys. They think donkeys don't need to be fed” (June 9 2014).

In contrast, many of the donkey owners spoke about the importance of providing their donkeys with additional feed on top of their grazing, particularly on markets days and other occasions when the donkey was required to work hard. They also mentioned that providing feed would increase the length of the donkey's working life. Some of those owners who did not provide feed recognized the need to do so but were unable to in practice; for example, Donkey owner number 15 explained that:

“I wish I could give additional feed to my donkeys. There is a grain shortage in the area” (June 19, 2014).

Key informant number 5, an urban shop owner, explained that “[people have a] very bad perception toward donkeys. They have no respect for donkeys they just use them” (June 12, 2014).

This statement was not supported by donkey owners, although positive regard for donkeys was often intertwined with their reliance on them and the income they generate. Donkey owner number 4 explained that:

“People love donkeys, they are depending on donkeys and no one dislikes donkeys” (June 9, 2014).

Donkey owner number 3, however, expressed positive views of donkeys beyond her reliance on them:

“I have heard that people insult each other by saying ‘donkey’ when they insult someone. But I know that donkeys are strong and brave and good so if someone insults me by calling me a donkey I will feel happy” (June 12, 2014).

The perception that donkey users lack respect for the animals they use was reiterated by other Key informants in the urban areas who claimed that the major welfare concerns affecting donkeys were not inadequate housing or risk of disease transmission but, rather, the lack of willingness on the part of owners/users to provide basic care for their donkeys. Despite earning sufficient income to provide the necessary food and veterinary care, the urban key informants lamented the donkey owners/users lack of willingness to maintain and improve their donkeys' health. Key informant number 3, a veterinary surgeon, explained:

“There has never been and there are no welfare standards for donkeys in this area [Addis Ababa]. People are not caring for their donkeys in this area. They are not treating their donkeys. People are not feeding their donkeys and they are earning more money, even more than people working with vehicles. People say working with donkeys is more profitable and they don't feed them, they just let them graze on the ground. But they are getting all this income from donkeys but they are not providing any care to them” (June 12, 2014).

This participant also explained that “[they] don't care at the end when the donkeys can't work anymore because they know they can easily replace that donkey because of what they are earning from the other donkeys so they don't care what happens to the donkey that can't work” (June 12, 2014).

This view was not expressed toward the donkey owners in rural areas suggesting significant differences in values and perceptions of donkeys between urban and rural locations.

Of the key informants, only the donkey trader, veterinary surgeons, development agent, and pharmacy owner generated direct income from donkey owners accessing their services such as their need for veterinary attention for injured or sick donkeys or the purchasing of a new donkey. The key informants who owned donkeys and made money directly from owning donkeys were only the donkey trader and the village chief. However, all the key informants had either personal experiences with donkeys or were able to provide opinions of donkeys and their role in wider Ethiopian society. Table 2 provides a list of the ten key informants interviewed and their respective occupations. The diversity of positions within society held by key informants generated rich accounts of data on the scale at which working donkeys contribute to communities, the roles they perform, and how they are perceived by wider society.

**Table 2:** Demographic information for the 10 key informants who participated in the study.

**Informant**	**Age range**	**Occupation**	**Location**	**Number of donkeys owned**
1	21–30	Health Extension Worker	Rural	0
2	21–30	Development Agent Animal Health Science	Rural	0
3	21–30	Veterinary Surgeon	Urban	0
4	51–60	Security Guard	Urban	0
5	21–30	Shop Owner	Urban	0
6	41–50	Donkey Trader	Rural	2
7	21–30	Chief of the village	Rural	4
8	21–30	Pharmacy Owner	Peri-urban	0
9	11–20	Non-donkey owner, unemployed	Peri-urban	0
10	21–30	Police Officer	Peri-urban	0

## Conclusion

The seven guiding themes of this paper have been donkeys as generators of income, the relationship between donkeys and social status, donkeys and affect, empowerment through donkeys, the role of donkeys in reducing vulnerability and encouraging resilience, donkey husbandry and gender dynamics, and differences between rural and urban settings all of which give a broader and richer insight into the value of donkeys. Interestingly, these themes can be applied to discussions of welfare for the donkey owners and the donkeys themselves. The interviews with both Key informants and Donkey owners/users yielded rich data on each of these themes, strongly indicating that together they form an effective framework upon which our tool to evaluate the socioeconomic value of donkeys can be created. The variations in views and practices between urban and rural settings suggests that assessing the socioeconomic value of donkeys within different locations within the same area or country is critical, rather than assuming that similar views are held between compatriots.

The focus of this study and ongoing project is directed on practices, beliefs and values within communities. However, it must be recognized that the donkeys within these communities are increasingly at risk from external threats. The recent rapid emergence of the donkey skin trade to meet global demand for the raw materials to make the traditional Chinese medicine ejiao, has had a significant impact on African donkeys. The trade has resulted in an escalating threat of donkey theft in many African countries (1, 29) and has had wide reaching consequences for donkey welfare and the livelihoods of some of those who depend upon them. Future studies on the implications of this global trade for the socioeconomic value of working donkeys within their communities is needed.

This paper demonstrates that the welfare of working donkeys and their owners/users is closely linked to the care humans receive, the social status and gender they are, and the level of economic vulnerability they have within the context in which they inhabit. Further investigation into the link between donkey welfare and human valuing is needed to explore to what extent donkey welfare is shaped by the socioeconomic values and beliefs attributed to them by humans.

## Data Availability Statement

The datasets generated for this study will not be made publicly available as the study participants did not explicitly give their consent for the full interview transcripts to be made publicly available.

## Ethics Statement

The study was approved by the Faculty of Medical and Veterinary Science Research Ethics Committee, University of Bristol (May 2014, Ref: 7502). Participant information sheets and consent forms were provided in the participant's first language of Amharic or Oromo and were also explained verbally in their first language to ensure comprehension prior to signing the consent form and starting the interview.

## Author Contributions

MGei, HB, FB, and HW conceptualized the study and its methodology. MGei, GT, and MGet conducted the study and collected the data. HW, FB, and HB supervised the study. MGei analyzed the data and wrote the original draft. JH analyzed the data and revised the original draft. All authors contributed to manuscript revision, read and approved the submitted version.

### Conflict of Interest

The authors declare that the research was conducted in the absence of any commercial or financial relationships that could be construed as a potential conflict of interest.
